# Coaxial Electrospun PLLA Fibers Modified with Water-Soluble Materials for Oligodendrocyte Myelination

**DOI:** 10.3390/polym13203595

**Published:** 2021-10-19

**Authors:** Zhepeng Liu, Jing Wang, Haini Chen, Guanyu Zhang, Zhuman Lv, Yijun Li, Shoujin Zhao, Wenlin Li

**Affiliations:** 1 School of Medical Instrument and Food Engineering, University of Shanghai for Science and Technology, Shanghai 200093, China; 202562371@st.usst.edu.cn (J.W.); 193832383@st.usst.edu.cn (H.C.); 172702210@st.usst.edu.cn (Y.L.); 183852335@st.usst.edu.cn (S.Z.); 2 Department of Cell Biology, Second Military Medical University, Shanghai 200433, China; zhangguanyu555@foxmail.com (G.Z.); lvzhuman@163.com (Z.L.)

**Keywords:** coaxial electrospinning, extracellular matrix, myelination, oligodendrocyte, water-soluble materials

## Abstract

Myelin sheaths are essential in maintaining the integrity of axons. Development of the platform for in vitro myelination would be especially useful for demyelinating disease modeling and drug screening. In this study, a fiber scaffold with a core–shell structure was prepared in one step by the coaxial electrospinning method. A high-molecular-weight polymer poly-L-lactic acid (PLLA) was used as the core, while the shell was a natural polymer material such as hyaluronic acid (HA), sodium alginate (SA), or chitosan (CS). The morphology, differential scanning calorimetry (DSC), Fourier transform infrared spectra (FTIR), contact angle, viability assay, and in vitro myelination by oligodendrocytes were characterized. The results showed that such fibers are bead-free and continuous, with an average size from 294 ± 53 to 390 ± 54 nm. The DSC and FTIR curves indicated no changes in the phase state of coaxial brackets. Hyaluronic acid/PLLA coaxial fibers had the minimum contact angle (53.1° ± 0.24°). Myelin sheaths were wrapped around a coaxial electrospun scaffold modified with water-soluble materials after a 14-day incubation. All results suggest that such a scaffold prepared by coaxial electrospinning potentially provides a novel platform for oligodendrocyte myelination.

## 1. Introduction

The myelin sheath wraps around the axons of neurons to provide protection, nutrition, and electrical insulation for axons [1]. Demyelinating diseases comprise a variety of disorders resulting from damage to oligodendrocytes, the myelin-forming cells, and consequent loss of myelin [2]. Demyelination could lead to devastating neurological impairments such as multiple sclerosis and cerebral palsy [3,4]. There are currently few effective therapies to regenerate the myelin [5,6]. The development of a platform for in vitro myelination would be highly useful for demyelinating disease modeling and drug screening [7,8]. Most studies have used a primary neuron and oligodendrocyte coculture system for an in vitro myelinating assay, which was time consuming and irreproducible [9]. Biocompatible polymers such as poly-L-lactic acid (PLLA), poly (lactic-co-glycolic) acid (PLGA), and poly (ε-caprolactone) (PCL) have been widely used as culture scaffolds to support cell proliferation and differentiation [10,11,12]. As the initiation of oligodendrocyte myelination does not depend on axonal signals [13], it is practicable to develop an artificial nanofiber scaffold for oligodendrocyte myelination. Previous efforts have used electrospun polystyrene or PLLA nanofibers as an artificial scaffold for oligodendrocyte myelination [14,15]. However, these scaffolds need to be coated with poly(l-lysine) to support cell attachment. Natural extracellular matrix (ECM) composition can provide biochemical and structural support for cell adhesion and regulate cell behaviors [16]. Scar formation is the biggest obstacle in the process of nerve regeneration. Studies have shown that hyaluronic acid (HA) can inhibit the generation of inflammation and promote the regeneration of nerve cells [17,18]. The non-antigenic nature of sodium alginate (SA) is more conducive to the repair of nerve cells [19]. SA gel was formed on the surface of the scaffold by the cross-linking method, which enhanced the biocompatibility of the scaffold and facilitated the proliferation and spread of cells on the scaffold surface [20]. Hossein et al. mixed chitosan (CS) particles with a scaffold to form a fibrous gel for sciatic nerve repair, and the results showed no significant difference in the sciatic nerve index compared with autograft [21]. However, most natural materials have high cell affinity and poor mechanical properties as cell scaffolds alone [22]. We reasoned that a coaxial stent structure with a water-soluble natural extracellular matrix outer layer and manmade polymer core could support better oligodendrocyte myelination. Therefore, in the present study, we aimed to develop a PLLA-based fiber scaffold with sodium hyaluronate, sodium alginate, or chitosan in the outer layer and to test their capacity to support myelination.

Common stent preparation methods include self-assembly [23,24], electrostatic spinning [25,26], and 3D printing [27,28], among others. The self-assembly method is an earlier method of preparation, and the process is simple and easy to operate. However, the self-assembled scaffold has weak mechanical properties, and it is difficult to create a stable three-dimensional structure, which results in the scaffold being unable to provide a stable place for cell growth and differentiation for a long time [29,30]. Three-dimensional (3D) printing is sought after by various industries due to its versatility and precision. However, the biological field requires far more resolution than most industries, resulting in slow printing processes and expensive equipment [31,32]. Electrospinning was used in the textile industry in its early days, but it has gradually expanded to many fields. This method has a relatively stable operation process and can produce uniform and continuous micron or nanofibers [33,34]. Currently, compared to electrospinning, many other stent preparation methods are relatively complicated to produce a suitable structure [35,36].

**Figure 1:**
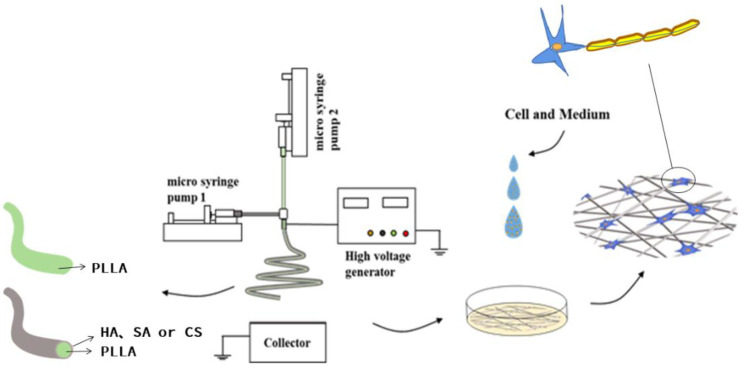
Coaxial electrospinning system.

We designed a coaxial electrospinning setup to prepare a coaxial stent intended to promote the myelination of oligodendrocyte. The stent contained sodium hyaluronate, sodium alginate, or chitosan in the outer layer and a PLLA core (as illustrated in Figure 1). These stent structures greatly enhanced oligodendrocyte myelination. As far as we know, this is the first report on a coaxial scaffold modified with natural water-soluble materials by electrospinning as preparation for the culture and myelination of human oligodendrocytes.

## 2. Materials and Methods

### 2.1. Materials

Sodium hyaluronate (HA, Mw = 1800 kDa) was purchased from the Bloomage Freda Biopharma Co., Ltd. (Jinan, China). Poly(L-lactic acid) (PLLA, Mw = 30 Kda) was obtained from Jinan Daigang Biomaterial Co., Ltd. (Jinan, China). Sodium alginate (SA, Mw = 270 kDa) and chitosan (CS, Mw = 500,000, viscosity between 200 and 400 cP) were provided by Sinopharm Group Shanghai Chemical Reagent Company (Shanghai, China). Ethanol, dichloromethane (DCM), dimethyl sulfoxide (DMSO), and acetic acid were offered from Shanghai Vita Co., Ltd. (Shanghai, China).

MTT (4,5-dimethylthiazole-2))-2,5-diphenyltetrazolium bromide and 2-(4-amidinophenyl)-6-indole carbamidine dihydrochloride (DAPI) were purchased from Beyotime Biological Technology Co., Ltd. (Guangzhou, China). Rat adrenal pheochromocytoma cells (PC-12) were derived from the Cell Bank of the Type Culture Collection of the Chinese Academy of Sciences (Shanghai, China). Human embryonic stem cells (hESCs, H1 line) were obtained from WiCell (Madison, WI, USA).

### 2.2. Coaxial Electrospinning

The coaxial electrospinning platform used in this experiment was self-built, as shown in Figure 1. The inner and outer layers of spinning solution were controlled by two peristaltic pumps (KDS100, Scientific, Holliston, MA, USA), which were connected by homemade coaxial needles. The voltage controlled by the high-voltage generator (ZGF 60, Huatian Power Automation Co., Ltd., Wuhan, China) was applied to the needle through an alligator clip. An aluminum foil collector was used as a receiving device.

Firstly, the optimum parameters of the electrospinning (solution concentration/flow rate/voltage/needle–collector distance) were investigated, and subsequently, the coaxial fibers were prepared using the optimized conditions. Briefly, we added 3 g of PLLA to 50 mL (DCM: DMSO, 9:1/v:v) solution as the core solution. We configured three different shell spinning solutions: 0.1 g of HA was dissolved in 10 mL of a 30% ethanol aqueous solution, 0.1 g SA was dissolved in 10 mL of water, and 0.1 g of CS was added to 10 mL of 75% acetic acid aqueous solution. Pure PLLA spinning was obtained at a spinning solution flow rate of 0.8 mL/h, a voltage of 14 KV, and a distance needle to collector of 10 cm. The core layer and shell layer spinning solution flow velocity of coaxial electrospinning were 0.8 mL/h, the applied voltage was adjusted within the range of 14–16 KV, and the distance needle to collector was within 10–12 cm. All electrospinning processes were carried out under ambient conditions (22 ± 3 °C with a relative humidity of 50 ± 5%).

### 2.3. Characterization

The fibers were characterized by size and appearance using an electron scanning microscope (SEM, Phenom ProX, Phenom, Eindhoven, The Netherlands). Before observing the fibers, the fibers were sprayed with gold. The diameter data of 100 random fibers in the photo were measured by ImageJ2x (Rawak Software Inc., Stuttgart, Germany), and the fiber diameter distribution was calculated to obtain the average fiber diameter. To visualize the core–sheath structure, transmission electron microscopy (Tecnai G^2^ F20 S-TWIN, Hillsboro, OR, USA) at an accelerating voltage of 200 kV was employed. The chemical structure of the fiber was analyzed by the Fourier transform infrared spectrometer (FT-IR, Nicolet iS 5, Thermo Fisher, Waltham, MA, USA) to assess whether the chemical structure of the fiber was changed before preparation. All FTIR spectra were obtained in the spectral region of 500–2500cm^−1^, with a resolution of 4 cm^−1^, after 20 scans of each sample. A differential scanning calorimeter (DSC, DSC 204, NETZSCH, Selb, Germany) was used for thermal analysis of the fibers. Approximately 5 mg of the sample was placed in a clean crucible and heated from 25 to 300 °C (heating rate of 10 °C/min and a nitrogen purge of 10 mL/min). The hydrophilicity and hydrophobicity of the fiber were judged by the contact angle detection (DSA30, Kruss, Hamburg, Germany). During the measurement, 0.03 mL of deionized water were dropped on the spun fiber, and each sample was measured five times and averaged.

### 2.4. Cell Culture

Human oligodendrocytes were derived from hESCs as reported with modification [37]. Briefly, hESCs were maintained with E8 medium on a Matrigel coated surface. To induce neural differentiation, hESCs were treated with 2 μM TGFβ inhibitor SB43142 (Selleck, Houston, TX, USA), 1 μM BMP inhibitor DMH1 (Selleck, Houston, TX, USA), and 100 nM retinoic acid (Sigma-Aldrich, St. Louis, MO, USA) for 7 days in DMEM/F12 media supplemented with N2 and B27; then, it was treated with 100 nM SMO agonist SAG (Selleck, Houston, TX, USA) and 100 nM retinoic acid for an additional 7 days. The differentiated cells were dissociated with Accutase into single cells and plated into ultra-low attachment plates (Corning) for suspension culture supplemented with 10 ng/mL PDGF-AA, 5 ng/mL HGF, 10 ng/mL IGF1, and 10 ng/mL NT3 for 40 days. The cell aggregates were dissociated with Accumax into single cells (cells could be frozen for future experiments at this time point) and seeded on cover glasses with different fiber scaffolds at a density of 1.0 × 10^4^ cells/cm^2^. The cells were cultured with DMEM/F12 media with N2, B27, 60 ng/mL T3 (Sigma-Aldrich, St. Louis, MO, USA), 100 ng/mL biotin (Sigma-Aldrich, St. Louis, MO, USA), 1 μM cAMP (Sigma-Aldrich, St. Louis, MO, USA), and 60 μg/mL ascorbic acid-2-phosphate (Sigma-Aldrich, St. Louis, MO, USA) for an additional 14–21 days.

Rat adrenal pheochromocytoma cells (PC-12) were cultured in an incubator at 37 °C and 5% CO_2_ concentration. The medium was a PC-12-defining medium (90% RPMI 1640 medium supplemented with 10% fetal bovine serum, 100 U/mL penicillin, and 100 μg/mL streptomycin). The medium was changed every two days. Prior to cell seeding, the four scaffolds prepared were placed in 24-well culture plates and UV-sterilized for 3 h. Cells were seeded on different scaffolds at a density of 1.0 × 10^4^ cells cm^2^.

All tissue culture products were obtained from Thermo Fisher Scientific except where otherwise specified.

### 2.5. Viability Assay

An MTT assay was used to evaluate the cytotoxicity of fiber scaffold to rat adrenal pheochromocytoma cells (PC-12). PC-12 were seeded onto different scaffolds at a density of 1.0 × 10^4^ per well of 96-well plates. After 12 h, 24 h, 48 h, and 72 h, the old medium was discarded and washed three times with prewarmed PBS. A total of 360 μL of the prewarmed culture medium and 40 μL of 5 mg/mL MTT solution were added to each well, and the culture was incubated for 4 h. Then, the medium was discarded, and 400 μL of DMSO was added to each well. After shaking in the dark at 37 °C for 30 min, the DMSO solution was transferred to a 96-well plate. The absorbance was measured with a microplate reader (MODEL 680, Bio-Rad, Hercules, CA, USA) at a wavelength of 492 nm.

### 2.6. In Vitro Oligodendrocyte Myelination

In this experiment, the myelination of oligodendrocytes on the scaffolds in each group was observed by microscope and immunofluorescence staining. The scaffolds cocultured with oligodendrocytes for 14 days were fixed with 4% paraformaldehyde for 5 min and then permeabilized with PBS buffer containing 0.5% Triton X-100 (Sigma-Aldrich, St. Louis, MO, USA) and 5% donkey serum (Jackson ImmunoResearch, West Grove, PA, USA) for 30 min at room temperature. The cells were incubated with rat antimyelin basic protein (MBP, Abcam, Cambridge, UK) and mouse anti-rat O4 antibody (R&D system, Minneapolis, MN, USA) at 4 °C overnight. Next, the cells were washed with PBST and incubated with Alexa Fluor 488 conjugated donkey anti-mouse IgM and Alexa Fluor 555 conjugated donkey anti-rat IgG secondary antibodies (Invitrogen, 1000×) in PBST for one hour at room temperature. Nuclei were visualized by DAPI staining. Images were captured using a fluorescence microscope (Nikon ECLIPSE Ti2, Tokyo, Japan).

### 2.7. Statistical Analysis

All data are expressed as the mean value ± SD. Statistical analysis was performed with one-way analysis of variance (ANOVA) in Graph Pad Prism 7 software. A *p*-value of less than 0.05 was considered statistically significant.

## 3. Results and Discussion

### 3.1. Morphology and Microstructure of the Scaffolds

**Figure 2:**
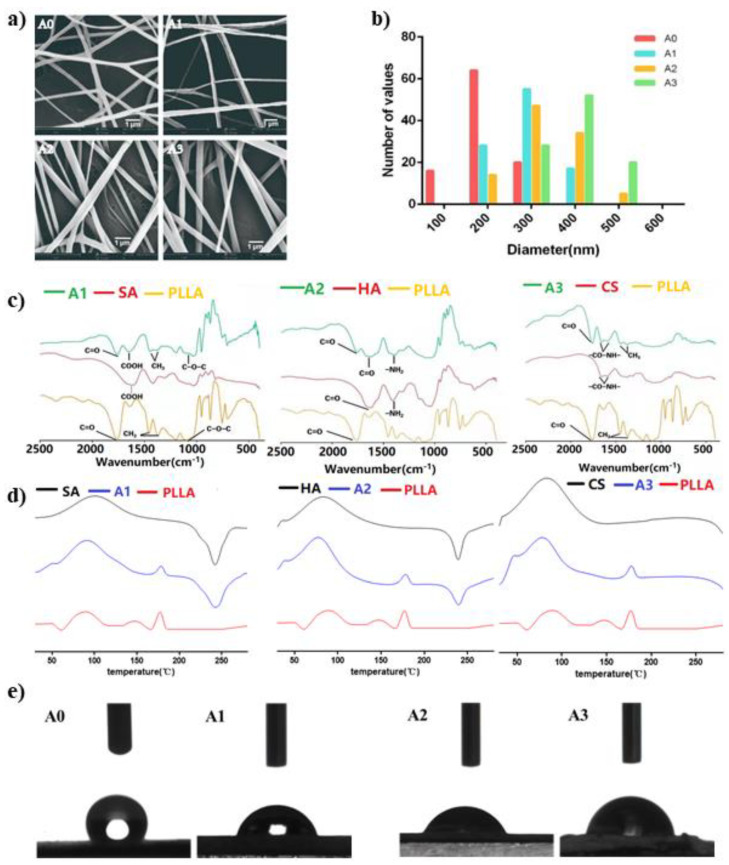
Physical and chemical properties investigation ((**a**,**b**) SEM of electrospinning fibers and diameter distribution; (**c**) Fourier transform infrared (FT-IR) of fiber scaffold; (**d**) DSC of fiber scaffold; (**e**) Experimental results of contact angles of spinning stent) (A0 6% PLLA; A1 1% sodium alginate (shell)—6% PLLA (core); A2 1% sodium hyaluronate (shell)—6% PLLA (core); A3 1% chitosan (shell)—6% PLLA (core)).

In order to compare the effects of different natural materials on the performance of fiber scaffolds, we prepared three different scaffolds. The shell materials were SA, HA, and CS. As shown in Figure 2a, all four fibers were bead-free and continuous. Lower magnitude SEM images are provided in Figure S1 (see Supplementary Materials). Among them, the pure PLLA spun was marked as A0, the spinning with SA as the shell was marked as A1, the outermost layer was HA spun as A2, and the outer layer of CS was labeled A3. It can be seen from Figure 2b that the diameter distribution of A0 fibers was relatively uniform, with an average diameter of 204 ± 44 nm. The diameter of the three types of coaxial electrospun fibers was basically larger than that of the A0. The smallest one was A1 with an average diameter of 294 ± 53 nm, and the largest was A3 with an average diameter of 390 ± 54 nm. This was due to the fact that the voltage and acceptance distance of A1 during the preparation process were larger than other groups. According to Maurya’s research results, the increase in voltage or the distance needle to collector within a certain range can refine the fiber diameter [38]. The spinning condition of group A2 was similar to that of group A3, and the average fiber diameter was 334 ± 69 nm. The average fiber diameter of coaxial nanofibers is much bigger than that of neat PLLA nanofibers. The high viscosity and vapor pressure of the shell solution may be the reasons for the increasing of coaxial fiber diameter compared to single-component nanofiber. A similar trend was reported by Afshar et al. [39] on the fabrication of coaxial electrospun CS/PLA fibers, which had bigger average diameters than neat PLA fibers. In another work [40], it was also shown that the diameters of coaxial (PVP/PLA) and mono (PLA) electrospun scaffolds were 599.9 ± 112.0 nm and 136.8 ± 10 nm, respectively. TEM studies (Figure S2, Supplementary Materials) revealed a successful formation of the core–sheath structure.

### 3.2. DSC and FT-IR

Interaction between scaffold materials can be detected by FT-IR (Figure 2c). On the pure PLLA fiber spectra, two peaks caused by C=O and C-O-C stretching vibration appeared at 1760 cm^−1^ and 1170 cm^−1^, which were consistent with the reports in the literature, and the absorption peaks at 1365 cm^−1^ and 1450 cm^−1^ were caused by CH_3_ [41,42]. HA was formed by the polymerization of glucuronic acid and acetaminohexose, 1612 cm^−1^ and 1412 cm^−1^, and the right corner of the valley was caused by the amide II [43,44]. The main functional groups in the SA molecule were carboxylate and glycosidic bonds, which corresponded to the characteristic peaks appearing at 1612 cm^−1^ and 1000–1150 cm^−1^ in the FT-IR spectrum [45]. Although CS is the product of deacetylation of chitin, the degree of deacetylation of CS used in the experiment was between 80% and 95%, so it still contained acyl groups. The absorption peaks at 1650 cm^−1^ and 1580 cm^−1^ in the spectrum were caused by acyl groups, while the glycosidic bonds were absorption peaks at 1100 cm^−1^ [44,46]. As expected, no new characteristic peaks appeared during the experiment, and the characteristic peaks of each component appeared in the spectrum of the coaxial bracket.

The DSC curves of different spun scaffolds are shown in Figure 2d. Compared with the other three materials, the pure PLLA showed an obvious endothermic peak at 178 °C. At the same time, this characteristic peak appeared in all coaxial bracket samples. The thermograms of SA and HA displayed an exothermic peak at about 236 °C and 240 °C, respectively, which were also observed in the coaxial fibers.

### 3.3. Hydrophilicity of the Different Coaxial Scaffolds

The contact angle of the liquid on the surface of the solid material is an important parameter to measure the wettability of the liquid on the surface of the material. As shown in Figure 2e, the contact angle of the pure PLLA fiber was 133° ± 0.45°, and the contact angles of the obviously coaxial electrospun scaffolds were 59.8° ± 0.36°, 53.1° ± 0.24°, and 77.3° ± 0.42°, among which the fibers of A2 had the smallest contact angle. This shows that the fiber surface is a natural material, which indirectly proves that the prepared spinning has a core–shell double-layer structure. Chang [47] et al. reported that the membranes prepared by hydrophilic materials reduced the contact angle and the membrane-containing HA had the smallest contact angle. In our study, A2 (HA) had the smallest contact angle.

### 3.4. In Vitro Cytotoxicity of the Different Coaxial Scaffolds

**Figure 3:**
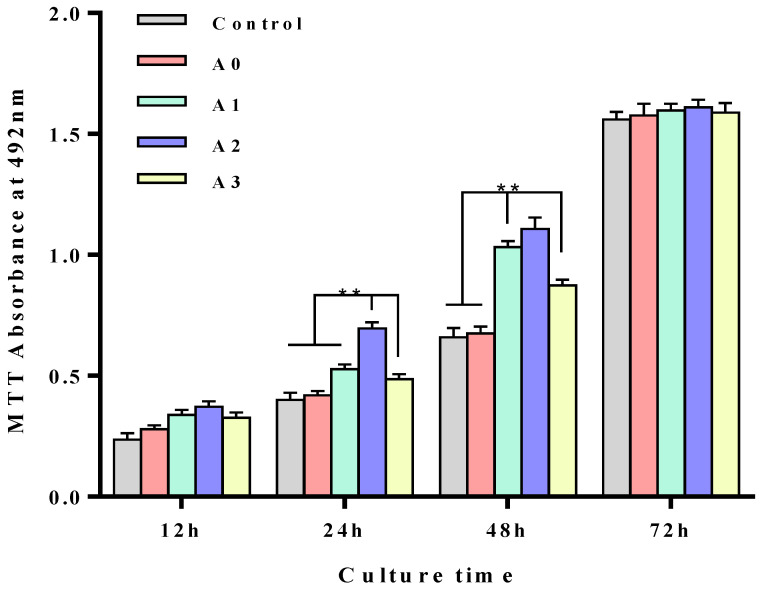
Cell viability of the different coaxial scaffolds. (Control) with no scaffold, (A0) 6% PLLA, (A1) 1% sodium alginate (shell)–6% PLLA (core), (A2) 1% sodium hyaluronate (shell)–6% PLLA (core), (A3) 1% chitosan (shell)–6% PLLA (core). * indicates *p* < 0.05, ** indicates *p* < 0.01.

To evaluate the quality of cell scaffolds, the first consideration is the cytotoxicity of the scaffolds. Due to the postmitotic nature of oligodendrocytes, PC-12 was selected as the target cell for cytotoxic assay. Through the MTT cell viability experiments, as shown in Figure 3, all the scaffolds could sustain PC-12 cell proliferation. Specifically, the coaxial stents with an HA (A2) soluble extracellular matrix outer layer demonstrated even better cell proliferation after 24 and 48-h incubation (*p* < 0.05). It was attributed to the best hydrophilic effect of A2, which had the smallest contact angle. However, as the time went on, PC-12 proliferation on the coaxial stents showed no significant difference. These data suggested that the prepared scaffold was not cytotoxic.

### 3.5. Oligodendrocytes Myelinate the Fiber Scaffolds

**Figure 4:**
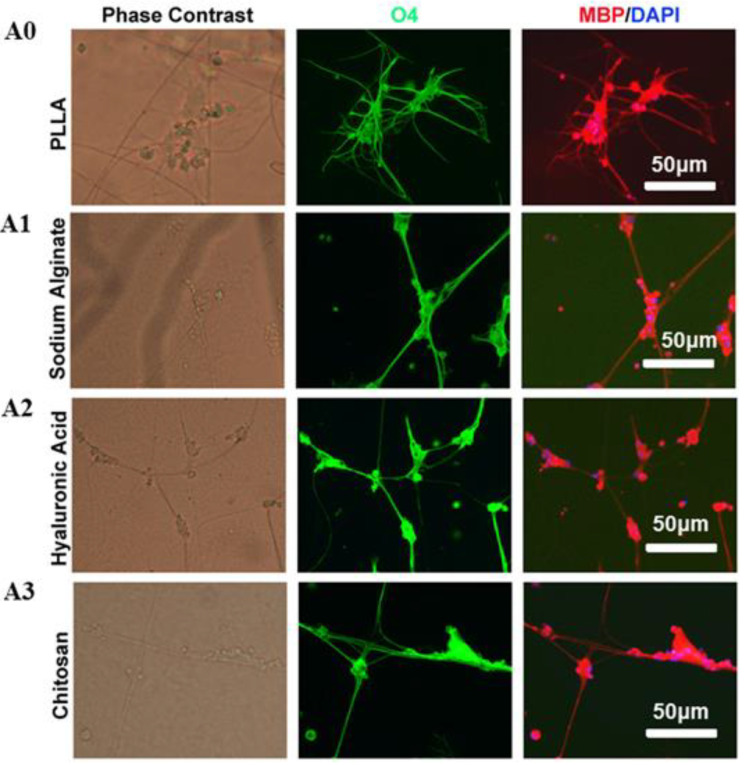
In vitro myelination of oligodendrocyte on different scaffolds after 14 days of cell seeding. Wrapped myelin positive for O4 and MBP was visualized by immunofluorescence staining. (A0) 6% PLLA, (A1) 1% sodium alginate (shell)–6% PLLA (core), (A2) 1% sodium hyaluronate (shell)–6% PLLA (core), (A3) 1% chitosan (shell)–6% PLLA (core).

After coculturing with different scaffolds for 14 days, the myelination of scaffolds by oligodendrocytes was analyzed by immunostaining. Oligodendrocytes formed MBP and O4 positive myelin sheaths along the fibers in all four tested scaffolds, which was consistent with the notion that oligodendrocytes can form myelin sheaths without the need of axonal signals. However, the MBP and O4 positive myelin sheaths were much longer in all three PLLA fibers modified with water-soluble materials than unmodified PLLA fibers, suggesting that the water-soluble matrixes used in this study, including sodium hyaluronate, sodium alginate, and chitosan, can enhance oligodendrocyte myelination (Figure 4). Makhijaa et al. reported that the stiffness, strain, topography, and spatial constraints of scaffolds play a key role in promoting the myelination of OPCs cells [48]. Hlavac et al. claimed that hydrogel scaffolds are suitable for nerve cell proliferation, differentiation, and repair [49]. In this study, PLLA scaffolds coated with hydrophilic material have played a very excellent role in promoting myelination.

## 4. Conclusions

In this experiment, the core–shell structured spinning scaffolds were prepared in one step by coaxial electrospinning technology for in vitro oligodendrocyte myelination. An MTT experiment showed that the scaffolds of each group were not cytotoxic. With 14 days of scaffold and oligodendrocyte coculturing, myelin sheaths were formed along the fibers. In particular, the fibers modified with water-soluble materials demonstrated longer myelin sheaths than unmodified PLLA fibers. These data suggested that these coaxial stents with a soluble natural extracellular matrix outer layer and synthetic polymer core could be better artificial scaffolds for oligodendrocyte myelination. This in vitro myelination culture system could be especially promising in screening candidates that can promote myelination for therapeutic purposes. However, although the initiation of myelination is an intrinsic property of oligodendrocytes, appropriate myelin compaction required an axon’s instructive signaling that is still not fully understood. Previous studies demonstrated that myelin sheaths wrapped around artificial fibers were aberrantly organized, which could represent a disadvantage of using artificial fibers as surrogates for neuron axons. Therefore, it is important to further characterize the myelin structures wrapped around these coaxial stents in the future. Our study provided a strategy to modify nanofibers for better in vitro oligodendrocyte myelination.

## Data Availability

Not applicable.

## Supplementary Materials

The following are available online at www.mdpi.com/10.3390/polym13203595/s1, Figure S1: SEM images of electrospinning fibers, Figure S2: TEM images of electrospinning fibers.

## References

[1] Stadelmann C., Timmler S., Barrantes-Freer A., Simons M. (2019). Myelin in the Central Nervous System: Structure, Function, and Pathology. Physiol. Rev..

[2] Love S. (2006). Demyelinating diseases. Clin. Pathol..

[3] Back S.A., Luo N.L., Borenstein N.S., Volpe J., Kinney H. (2002). Arrested oligodendrocyte lineage progression during human cerebral white matter development: Dissociation between the timing of progenitor differentiation and myelinogenesis. Neuropathol. Exp. Neurol..

[4] Compston A., Coles A. (2008). Multiple sclerosis. Lancet.

[5] Qi Z.P., Zhang T.H., Kong W.J., Fu C., Chang Y.X., Li H.R., Yang X.Y., Pan S. (2022). A dual-drug enhanced injectable hydrogel incorporated with neural stem cells for combination therapy in spinal cord injury. Chem. Eng. J..

[6] Nemeth C.L., Fine A.S., Fatemi A. (2019). Translational challenges in advancing regenerative therapy for treating neurological disorders using nanotechnology. Adv. Drug Deliv. Rev..

[7] Mozafari S., Evercooren A.B.V. (2021). Human stem cell-derived oligodendrocytes: From humanized animal models to cell therapy in myelin diseases. Semin. Cell Dev. Biol..

[8] Catherine L., Bernard Z., Anna W., Christine S., Bruno S. (2020). Remyelination in multiple sclerosis: From basic science to clinical translation. Lancet Neurol..

[9] Rodrigues G., Gaj T., Adil M., Wahba J., Rao A.T., Lorbeer F.K., Kulkarni R.U., Diogo M.M., Cabral J., Miller E.W. (2017). Defined and Scalable Differentiation of Human Oligodendrocyte Precursors from Pluripotent Stem Cells in a 3D Culture System. Stem Cell Rep..

[10] Parinaz A., Fatemeh O., Ahad M. (2021). The triad of nanotechnology, cell signalling, and scaffold implantation for the successful repair of damaged organs: An overview on soft-tissue engineering. J. Control. Release.

[11] Luo Y.Q., Xue F., Liu K., Li B.Q., Fu C.F., Ding J.X. (2021). Physical and biological engineering of polymer scaffolds to potentiate repair of spinal cord injury. Mater. Des..

[12] Mneimneh A.T., Mehanna M.M. (2021). Collagen-based scaffolds: An auspicious tool to support repair, recovery, and regeneration post spinal cord injury. Int. J. Pharm..

[13] Rosenberg S.S., Kelland E.E., Tokar E., De la Torre A.R., Chan J.R. (2008). The geometric and spatial constraints of the microenvironment induce oligodendrocyte differentiation. Proc. Natl. Acad. Sci. USA.

[14] Nocita E., Giovane D.A., Tiberi M., Boccuni L., Fiorelli D., Sposato C., Romano E., Basoli F., Trombetta M., Rainer A. (2019). EGFR/ErbB Inhibition Promotes OPC Maturation up to Axon Engagement by Co-Regulating PIP2 and MBP. Cells.

[15] Nathalie B., Ana M., Sandra V., Abílio A., Maria H.V.F., Paula M.V., Odete A.B.D.C. (2018). Electrically polarized PLLA nanofibers as neural tissue engineering scaffolds with improved neuritogenesis. Colloids Surf. B Biointerfaces.

[16] Marie C., Pierre J., Onnik A., Christophe H. (2022). 3D models of dilated cardiomyopathy: Shaping the chemical, physical and topographical properties of biomaterials to mimic the cardiac extracellular matrix. Bioact. Mater..

[17] Kumar G.S., Murugakoothan P. (2014). Synthesis, spectral analysis, optical and thermal properties of new organic NLO crystal: N,N′-Diphenylguanidinium Nitrate (DPGN). Spectrochim. Acta Part A Mol. Biomol. Spectrosc..

[18] Wang X., He J., Wang Y., Cui F.Z. (2012). Hyaluronic acid-based scaffold for central neural tissue engineering. Interface Focus.

[19] Wu Z., Li Q., Xie S., Shan X., Cai Z. (2020). In vitro and in vivo biocompatibility evaluation of a 3D bioprinted gelatin-sodium alginate/rat Schwann-cell scaffold. Mater. Sci. Eng. C.

[20] Homaeigohar S., Tsai T.Y., Young T.H., Yang H.J., Ji Y.R. (2019). An electroactive alginate hydrogel nanocomposite reinforced by functionalized graphite nanofilaments for neural tissue engineering. Carbohydr. Polym..

[21] Jahromi H.K., Farzin A., Hasanzadeh E., Barough S.E., Mahmoodi N., Najafabadi M.R.Z., Farahani M.S., Mansoor K., Shirian S., Ai J. (2020). Enhanced sciatic nerve regeneration by poly-L-lactic acid/multi-wall carbon nanotube neural guidance conduit containing Schwann cells and curcumin encapsulated chitosan nanoparticles in rat. Mater. Sci. Eng. C.

[22] Rajasekaran R., Seesala V.S., Sunka K.C., Ray P.G., Saha B., Banerjee M., Dhara S. (2020). Role of nanofibers on MSCs fate: Influence of fiber morphologies, compositions and external stimuli. Mater. Sci. Eng. C.

[23] Negah S.S., Oliazadeh P., Jahan-Abad A.J., Eshaghabadi A., Samini F., Ghasemi S., Asghari A., Gorji A. (2019). Transplantation of human meningioma stem cells loaded on a self-assembling peptide nanoscaffold containing IKVAV improves traumatic brain injury in rats. Acta Biomater..

[24] Sarode A., Annapragada A., Guo J.L., Mitragotri S. (2020). Layered self-assemblies for controlled drug delivery: A translational overview. Biomaterials.

[25] Wang Z., Wang Y.C., Yan J.Q., Zhang K.S., Lin F., Xiang L., Deng L.F., Guan Z.P., Cui W.G., Zhang H.B. (2021). Pharmaceutical electrospinning and 3D printing scaffold design for bone regeneration. Adv. Drug Deliv. Rev..

[26] Mokhtari F., Azimi B., Salehi M., Hashemikia S., Danti S. (2021). Recent advances of polymer-based piezoelectric composites for biomedical applications. J. Mech. Behav. Biomed. Mater..

[27] Xu W.H., Jambhulkar S., Zhu Y.X., Ravichandran D., Kakarla M., Vernon B., Lott D.G., Cornella J.L., Shefi O., Miquelard-Garnier G. (2021). 3D printing for polymer/particle-based processing: A review. Compos. Part B Eng..

[28] Vijayavenkataraman S., Thaharah S., Zhang S., Lu W.F., Fuh J.Y.H. (2019). Electrohydrodynamic jet 3D-printed PCL/PAA conductive scaffolds with tunable biodegradability as nerve guide conduits (NGCs) for peripheral nerve injury repair. Mater. Des..

[29] Stuart K., Amalia A., Eileen L., McPherson M.J. (2009). Production of self-assembling biomaterials for tissue engineering. Trends Biotechnol..

[30] Koss K.M., Unsworth L.D. (2016). Neural tissue engineering: Bioresponsive nanoscaffolds using engineered self-assembling peptides. Acta Biomater..

[31] Ji S.C., Kang H.-W., Lee I.H., Ko T.J., Cho D.-W. (2009). Development of micro-stereolithography technology using a UV lamp and optical fiber. Int. J. Adv. Manuf. Technol..

[32] O’Brien C.M., Holmes B., Scott F., Zhang L.J.G. (2015). Three-Dimensional Printing of Nanomaterial Scaffolds for Complex Tissue Regeneration. Tissue Eng. Part B Rev..

[33] Jain R., Shetty S.S., Yadav K. (2020). Unfolding the electrospinning potential of biopolymers for preparation of nanofibers. J. Drug Deliv. Sci. Technol..

[34] Ghosal K., Agatemor C., Špitálsky Z., Thomas S., Kny E. (2019). Electrospinning tissue engineering and wound dressing scaffolds from polymer-titanium dioxide nanocomposites. Chem. Eng. J..

[35] Ha D.H., Chae S.H., Lee J.Y., Kim J.Y., Yoon J.B., Sen T., Lee S.W., Kim H.J., Cho J.H., Cho D.W. (2021). Therapeutic effect of decellularized extracellular matrix-based hydrogel for radiation esophagitis by 3D printed esophageal stent. Biomaterials.

[36] Kumar R., Aadil K.R., Ran J.S., Vijay B.K. (2020). Advances in nanotechnology and nanomaterials based strategies for neural tissue engineering. J. Drug Deliv. Sci. Technol..

[37] Douvaras P., Fossati V. (2015). Generation and isolation of oligodendrocyte progenitor cells from human pluripotent stem cells. Nat. Protoc..

[38] Maurya A.K., Narayana P.L., GeethaBhavani A., Hong J.K., Reddy N.S. (2020). Modeling the relationship between electrospinning process parameters and ferrofluid/polyvinyl alcohol magnetic nanofiber diameter by artificial neural networks. J. Electrost..

[39] Afshar S., Rashedi S., Nazockdast H., Ghazaliand M. (2019). Preparation and characterization of electrospun poly(lactic acid)-chitosan core-shell nanofibers with a new solvent system. Int. J. Biol. Macromol..

[40] Hajikhani M., Emam-Djomeh Z., Askari G. (2021). Fabrication and characterization of mucoadhesive bioplastic patch via coaxial polylactic acid (PLA) based electrospun nanofibers with antimicrobial and wound healing application. Int. J. Biol. Macromol..

[41] Bonadies I., Longo A., Androsch R., Jehnichen D., Göbel M., Lorenzo M.L.D. (2019). Biodegradable electrospun PLLA fibers containing the mosquito-repellent DEET. Eur. Polym. J..

[42] Liu Z.X., Yang Y., Zhang K. (2013). Control of structure and morphology of highly aligned PLLA ultrafine fibers via linear-jet electrospinning. Polymer.

[43] Safaei M., Taran M. (2017). Optimal conditions for producing bactericidal sodium hyaluronate-TiO2 bionanocomposite and its characterization. Int. J. Biol. Macromol..

[44] Coimbra P., Alves P., Valente T.A., Santos R., Correia I.J., Ferreira P. (2011). Sodium hyaluronate/chitosan polyelectrolyte complex scaffolds for dental pulp regeneration: Synthesis and characterization. Int. J. Biol. Macromol..

[45] Salem D., Sallam M.A.E., Youssef T. (2019). Synthesis of compounds having antimicrobial activity from alginate. Bioorg. Chem..

[46] Mauricio A., Salazar R., Luna-Bárcenas G., Mendoza-Galvan A. (2018). FTIR spectroscopy studies on the spontaneous neutralization of chitosan acetate films by moisture conditioning. Vib. Spectrosc..

[47] Chang P.H., Chao H.M., Chern E., Hsu S.H. (2021). Chitosan 3D cell culture system promotes naïve-like features of human induced pluripotent stem cells: A novel tool to sustain pluripotency and facilitate differentiation. Biomaterials.

[48] Makhijaa E.P., Espinosa-Hoyos D., Jagielska A., Van Vliet K.J. (2020). Mechanical regulation of oligodendrocyte biology. Neurosci. Lett..

[49] Hlavac N., Kasper M., Schmidt C.E. (2020). Progress toward finding the perfect match: Hydrogels for treatment of central nervous system injury. Mater. Today Adv..

