# Supplementary material for: Coaxial Electrospun PLLA Fibers Modified with Water-Soluble Materials for Oligodendrocyte Myelination
Source: Polymers (Basel). 2021 Oct 19;13(20):3595. doi: 10.3390/polym13203595 (PMC8537353; doi:10.3390/polym13203595)
Supplement: Supplementary file 1 [file polymers-13-03595-s001.zip › polymers-1386016-supplementary.pdf]

Coaxial Electrospun PLLA Fibers Modified with Water-Soluble Materials for Oligodendrocyte Myelination

Zhepeng Liu [1,*], Jing Wang [1], Haini Chen [1], Guanyu Zhang [2], Zhuman Lv [2], Yijun Li [1], Shoujin Zhao [1] and Wenlin Li [2,*]

[1] School of Medical Instrument and Food Engineering, University of Shanghai for Science and Technology, Shanghai 200093, China
[2] Department of Cell Biology, Second Military Medical University, Shanghai 200433, China
* Correspondence: zpliu@usst.edu.cn (Z.L.); liwenlin@smmu.edu.cn (W.L.)

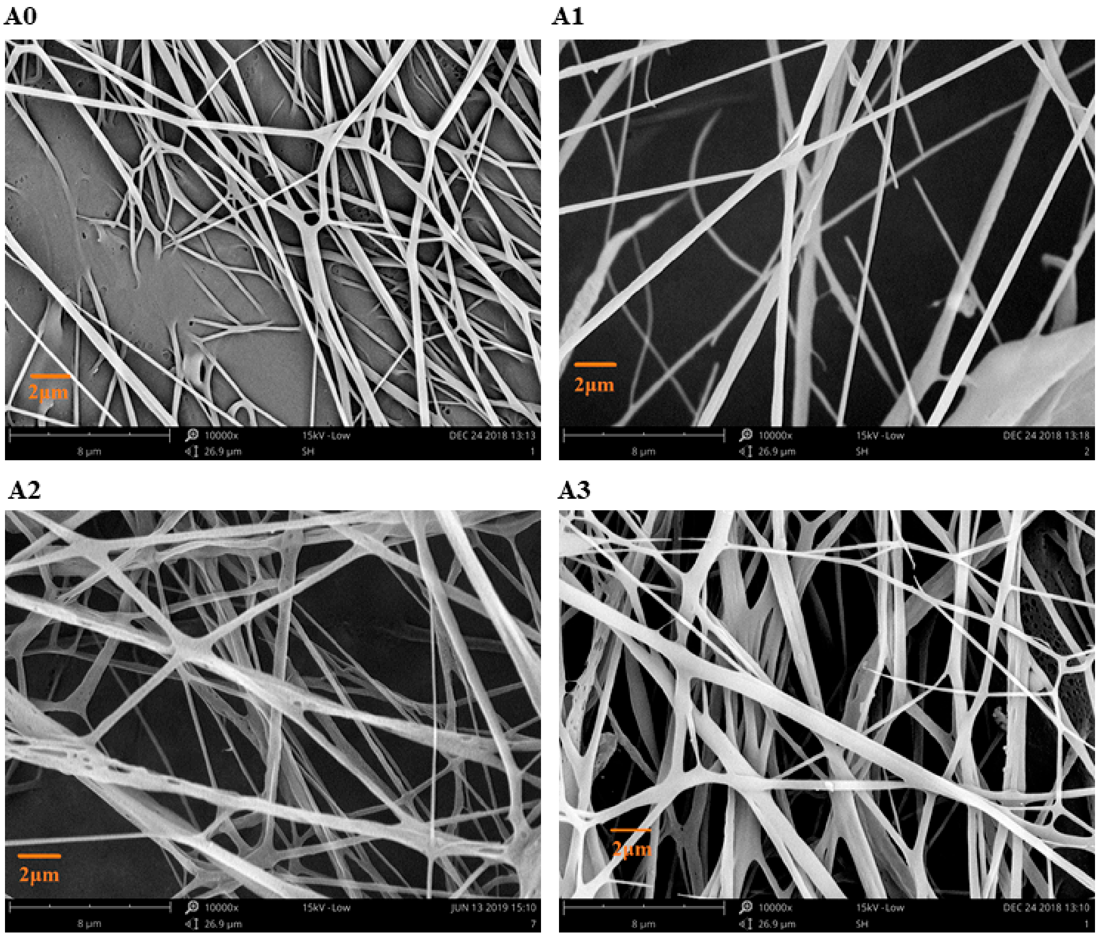

Figure S1. SEM images of electrospinning fibers. A0: 6% PLLA, A1: 1% sodium alginate (shell) – 6% PLLA (core), A2: 1% sodium hyaluronate (shell) – 6% PLLA (core), A3: 1% chitosan (shell) – 6% PLLA (core).

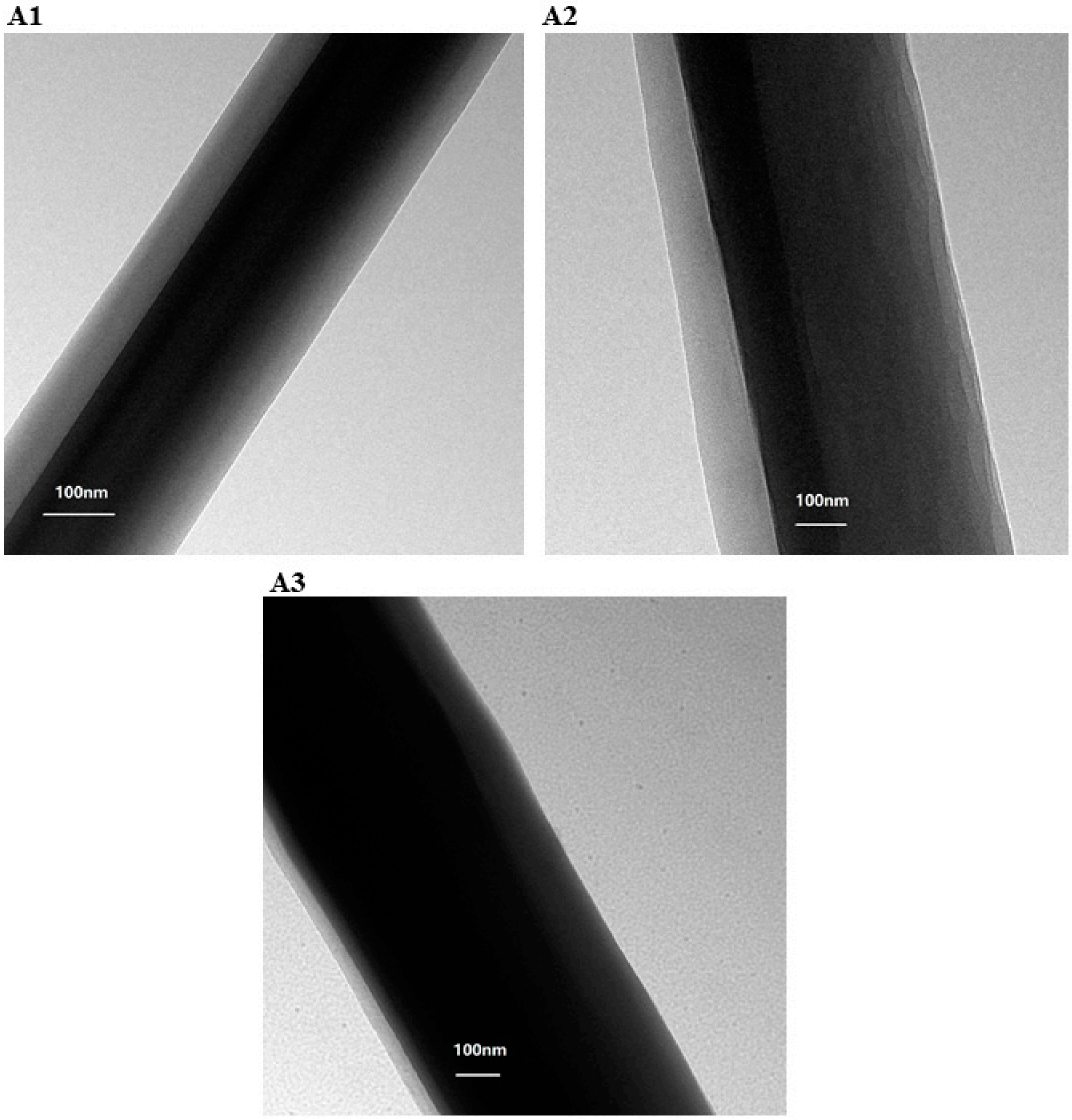

Figure S2. TEM images of electrospinning fibers. A1: 1% sodium alginate (shell) – 6% PLLA (core), A2: 1% sodium hyaluronate (shell) – 6% PLLA (core), A3: 1% chitosan (shell) – 6% PLLA (core).
